# HIV Vaccine Research: The Challenge and the Way Forward

**DOI:** 10.1155/2015/503978

**Published:** 2015-03-15

**Authors:** Hai-Bo Wang, Qiu-Hua Mo, Ze Yang

**Affiliations:** Zhuhai International Travel Healthcare Center, Zhuhai Entry-Exit Inspection and Quarantine Bureau, Zhuhai, Guangdong 519020, China

## Abstract

Human immunodeficiency virus/acquired immune deficiency syndrome (HIV/AIDS) is a worldwide epidemic, with over 35 million people infected currently. Therefore, the development of a safe and effective HIV-1 vaccine is on top of the global health priority. In the past few years, there have been many promising advances in the prevention of HIV/AIDS, among which the RV144 Thai trial has been encouraging and suggests optimization of the current vaccine strategies or search for novel strategies. Here we reviewed the brief history of HIV-1 vaccine, analyzed key challenges existing now, and illustrated future research priority/directions for a therapeutic or prophylactic HIV-1 vaccine, with the hope of accelerating the speed of vaccine development. We believe that an effective HIV-1 vaccine, together with other prevention approaches, will bring an end to this epidemic in the near future.

## 1. Introduction

Over thirty years have passed since the discovery of HIV as the causative agent of AIDS by Sinoussi et al. and Gallo et al. in 1983 [1, 2]. Now there are more than 35 million people living with HIV and 25 million individuals died of it. In 2013, over 5700 people become newly infected with HIV every day [3]. Although current highly active antiretroviral therapy (HAART) allows viral replication to be controlled [4–6], HIV-1 has not been eliminated. Latent reservoir, characterized by latently infected resting memory CD4+ T-cells, existed. Therefore, HIV cure is not possible until this reservoir is purged [7]. Moreover, there are about 90% of the infected population residing in developing countries [3] where antiretroviral drugs are generally not available. Therefore, the development of a safe and effective prophylactic HIV-1 vaccine would be the best for the ultimate elimination of the AIDS pandemic. However, no fully effective HIV vaccine is available till now.

## 2. Brief History of HIV Vaccine

Since the first phase I human trial of AIDS vaccine in 1986 in Zaire (now the Democratic Republic of Congo) by Zagury et al. [8], more than 250 clinical trials had been conducted, most of which were early-phase trials (phase I or II) [9]. Normally, neutralizing antibodies were the first choice for vaccine-induced immunity against infectious diseases, such as yellow fever and HIV-1. Therefore, lots of the researches in the first 10 years focused on humoral anti-HIV immunity [10]. Based on this concept, scientists used monomeric HIV-1 Env gp120 protein to induce Env-specific humoral immune responses. In early-phase clinical trials, though gp120 immunogens could elicit type-specific binding antibodies to the immunogens themselves, they failed to induce broadly neutralizing antibodies (bNAbs). In two phase III efficacy trials sponsored by VaxGen, the vaccine candidates afforded no detectable protective efficacy, indicating that these type-specific antibody responses did not provide protection against HIV-1 infection in humans [11, 12].

In 1994, the fact that the antibody-inducing HIV vaccines failed to provide protection against HIV-1 infection in humans called for a reevaluation of the global vaccine effort, which led scientists to cellular immune response. Evidence for cellular immune protection came from early vaccine studies conducted in rhesus monkeys by Hirsch et al. [13] and Shiver et al. [14]. In their study, no sterile protection was observed; however, they indeed observed prolonged survival of rhesus monkeys after homologous SIV challenge, and this appeared to be correlated with a lower viral set point. Virus-specific T lymphocyte responses seemed to play a critical role in controlling SIV replication and, therefore, the field shifted to T-cell immunity. The most famous HIV-1 vaccine focused on T-cell immunity is HIV Vaccine Trials Network (HVTN) 502, also known as the “STEP” trial, which was initiated by Merck and the National Institutes of Health to determine whether HIV-1-specific T-cell immunity induced by this vaccine could provide prevention from HIV-1 infection or at least would reduce plasma viral loads after infection. The vaccine candidate was formulated as a trivalent mixture of rAd5 vectors expressing HIV-1 clade B Gag, Pol, and Nef, respectively. Preclinical and phase I trial showed that this vaccine was highly immunogenic and could reduce viral loads after challenge of rhesus monkeys with the chimeric simian-human immunodeficiency virus- (SHIV-) 89.6P [14–16]. However, STEP trial was terminated on 18 September 2007 unexpectedly [17]. The vaccine could neither prevent infection nor decrease early plasma virus levels in those who received the vaccine, compared to the placebo recipients [18]. Moreover, a completely unexpected observation emerged in the STEP trial, in which a greater number of vaccine recipients got infected [19].

Surprisingly, two years after the failure of STEP trial, the RV144 Thai trial demonstrated a 31.2% efficacy in preventing HIV-1 infection [20], which was the first vaccine showing a modest protection. The trial used a “prime-boost” combination of two vaccines including vCP1521 canarypox vectored vaccine, which was manufactured by Sanofi Pasteur, and AIDSVAX B/E gp120 subunit vaccine, which was previously tested in the VAX003 and VAX004 trial. The immune correlates analysis of this trial indicated that V1V2 antibodies may have contributed to the protection against HIV-1 infection, whereas high levels of Env-specific IgA antibodies may have mitigated the effects of protective antibodies [21]. The analysis failed to identify neutralization antibody as a potential correlate, but, surprisingly, the nonneutralizing antibodies, especially those involved in mediating antibody-dependent cell-mediated cytotoxicity (ADCC), may play a role in the protection. The author suggested that future vaccines that are designed to induce higher levels of V1V2 antibodies and lower levels of Env-specific IgA antibodies than the RV144 vaccine may exhibit stronger efficacy against HIV-1 infection.

## 3. Challenge before Us

After the RV144 trial, the scientific community realized that an effective HIV vaccine would be achievable as long as we could learn from the past, figure out the key challenge before us, and explore novel vaccine concepts. However, to a great extent, HIV-1 vaccine research is still inchoate and there remain many unsolved problems [22]. Firstly, the extensive viral subtype and sequence diversity may be the greatest block to a broad HIV vaccine [23]. The HIV reverse transcriptase was an “error-prone” enzyme so huge diverse and constantly evolving virus populations could be generated within infected individuals [24]. Even within a particular subtype, the amino acid sequences of Env could differ up to 20% while the difference could reach up to 35% between subtypes [23, 24]. Secondly, the HIV-1 envelope glycoprotein was a trimer expressed on the surface of HIV-1 virion and contained extensive N-linked glycosylation which effectively shielded conserved epitopes from antibody recognition [25, 26]. Moreover, some conserved regions, such as the coreceptor binding site, were only formed after Env binding to CD4 molecular and undergoing an extensive conformational change [27]. In addition, the few bNAbs isolated from infected individuals seemed to require extensive somatic hypermutation of antibody genes [28]. Thirdly, there was propensity of the virus to accumulate mutations in T lymphocyte epitopes and then to evade cellular immune control [29–31]. And, in some instance, vaccine-induced T-cell immunity may increase the chances of infection, as shown by the data from the STEP trial [19]. Other challenges laid in unclear immune correlates of protection, lack of a relevant animal model, and perhaps little pharmaceutical interest [32].

## 4. The Way Forward

Despite the effective antiretroviral treatments, a therapeutic or prophylactic HIV vaccine still remains to be of vital importance, which needs all scientists' continuous effort. Traditional vaccine technologies included live attenuated viruses [33], whole killed viruses [34], and protein subunits vaccine [11]. However, the safety concerns [35] and inability to induce bNAbs [11, 12] limited their wide usage in HIV vaccine. Therefore, researchers now pay more attention to some novel vaccine strategies. For example, to address the genetic diversity of globally circulating strains of HIV-1, the consensus [23] or mosaic [36] immunogens, engineered by* in silico* analysis of global HIV-1 sequences to provide maximal coverage of viral sequence diversity, were designed. Gao et al. showed that the computer-generated “consensus” Env genes were capable of expressing envelope glycoproteins which retained the structural, functional, and immunogenic properties of wild-type HIV-1 envelopes and induced antibodies which modestly neutralized selected HIV-1 primary isolates [37]. Results from Liao et al. indicated that consensus Env elicited more potent responses in guinea pigs than Env from transmitted/founder virus or chronic viruses. The antibody induced could even neutralize some of the more-difficult-to-neutralize tier-2 viruses [38]. For mosaic vaccines, several groups showed that mosaic immunogens induced CD8+ T lymphocyte responses with extended breadth and depth in nonhuman primates [39, 40]. More importantly, Barouch et al. showed that a global HIV-1 mosaic vaccine elicited protective immune responses against heterologous SHIV challenges in rhesus monkeys [41], which was considered the first step toward a globally effective HIV/AIDS vaccine [42]. These advances made the broad protective vaccine more promising.

Given the unusual structure properties of bNAbs (high somatic mutation and long CDRH3) which could not be induced by traditional vaccination approaches, alternatives will be required. The first one is sequential vaccination. By studying the coevolution of the HIV virus and antibodies in an African donor who developed neutralizing antibodies, Liao et al. demonstrated that the virus evolution was concomitant with antibody maturation. Moreover, they showed that the evolution of antibody neutralization breadth was preceded by extensive viral diversification in and near the CH103 epitope [43]. These data indicated that the virus and antibody coevolution led to induction of a lineage of HIV-1 broadly neutralizing antibodies and provided insights into strategies to elicit similar neutralization antibodies by mimicking HIV envelope evolution* via* sequential vaccination. The second approach is to design immunogens which could specifically activate B cells expressing the germline antibodies. Wild-type Env lacked detectable affinity for predicted germline precursors of bNAbs, making them poor immunogens to prime bNAb response. However, through engineering or* in silico* design, two groups showed that their designed immunogens were able to bind to and activate germline BCRs [44, 45]. These immunogens may not directly induce bNAbs, but they could serve as a promising prime vaccine to initiate the process of antibody-affinity maturation and to make bNAbs possible.

In addition, Sodora et al. believed that HIV vaccine research could greatly benefit from the study of SIV infections of natural hosts, such as sooty mangabeys, African green monkeys, and mandrills, which shared many features of HIV infection of humans [46]. However, these natural hosts usually did not develop immunodeficiency. So studies of SIV-infected natural hosts would provide a number of insights for the design of future new vaccine approaches. One potential new vaccine approach that might originate from it was to include components or adjuvants to decrease the availability of target cells for the virus at the level of mucosal tissues since natural SIV hosts expressed lower amounts of CCR5 on CD4+ T-cells. Another potential approach was to reduce the level of chronic immune activation in the event of breakthrough infection. In natural SIV infection, elevated immune activation in acute infection was rapidly downregulated during chronic infection. So if we could identify the mechanisms responsible for it, such as immunomodulatory pathways (T regulatory cells, the negative regulator PD-1, transforming growth factor-*β*, etc.) or specific virus proteins (Nef, Vpu, and Env), then we could develop immunogens that contained built-in factors to prevent the development of chronic immune activation in the event of HIV infection. Besides these, now scientists began to pay more attention to innate immunity. Innate immune systems provided immediate defense against infection [47] and were critical for shaping vaccine-elicited adaptive immune responses. Understanding how innate immunity regulated adaptive immune responses would be of vital importance for optimizing vaccine candidates [48].

Last but not least, one major aspect of HIV vaccine development was to identify correlates of protection. Though a number of vaccines had been tested in nonhuman primates and some of them claimed that correlates of protection were identified, whether these findings could be translated to human and were also correlates of protection from HIV acquisition or infection in humans remained unknown [49]. For human clinical trials, only five of them were advanced to phase III till now, with four disappointing results [11, 12, 17, 50] and one modest result [20]. And even this modest RV144 trial was challenged by the recent statistical analysis, which indicated that the vaccination had low-level efficacy, with more than or equal to 22% chance for no efficacy at all [51]. Therefore, more human clinical efficacy trials should be conducted as this was the only way to determine which strategies would provide optimal protection against HIV-1 in humans [52]. To promote correlates of protection discovery, Day and Kublin advocated that future clinical trial designs should consider whether enough breakthrough infections would occur in the vaccine arm to provide adequate power for correlates evaluation in the event of partial vaccine efficacy. Furthermore, designs which aimed to provide earlier efficacy evaluations and/or simultaneously evaluation for several regimens should be encouraged [53].

## 5. Conclusion

HIV/AIDS has presented unparalleled scientific, medical, and moral challenges to human beings since 1983. Although there have been multiple setbacks and obstacles in the road to an HIV vaccine, significant progress has been made in the past few years. By assessing what had occurred in the past and identifying what is the main challenge before us, we believe we will find the way out and eventually conquer HIV/AIDS and put the pandemic to an end.
